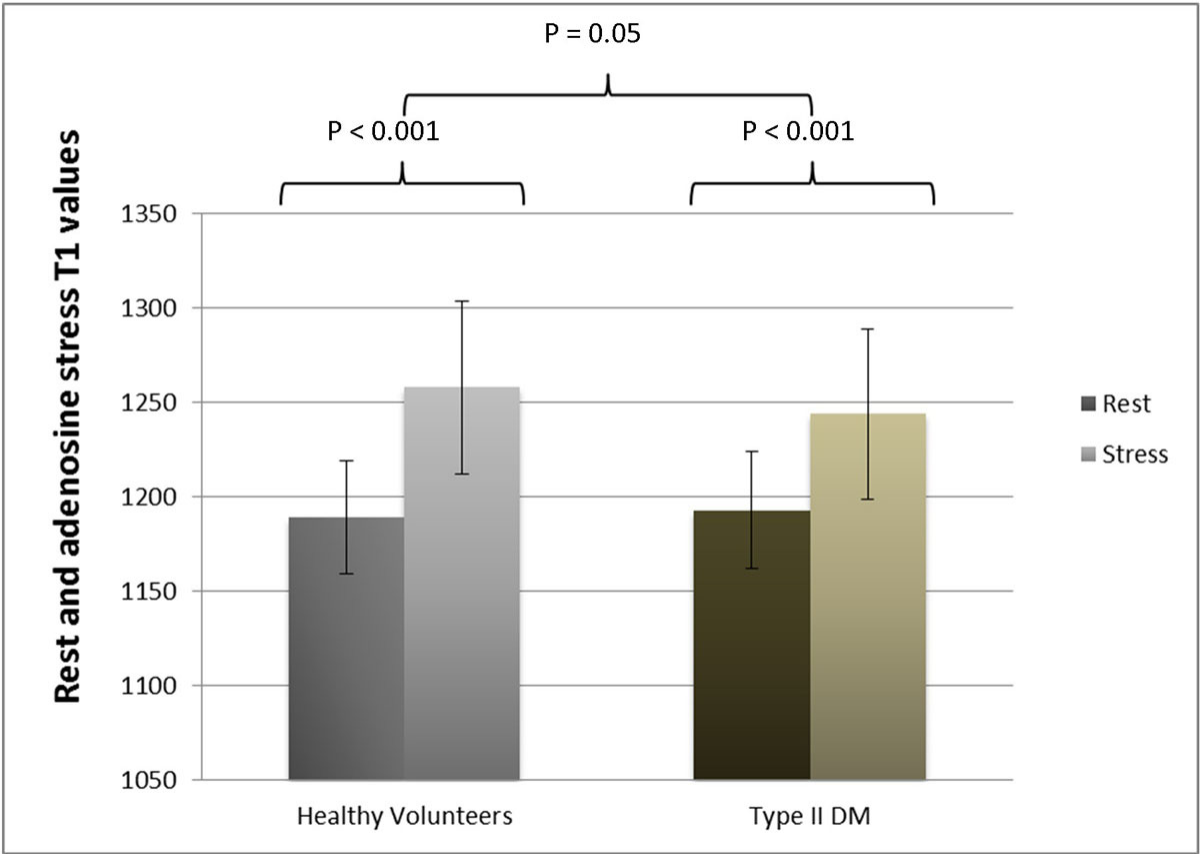# Adenosine stress native T1 mapping detects microvascular disease in diabetic cardiomyopathy, without the need for gadolinium-based contrast

**DOI:** 10.1186/1532-429X-17-S1-Q55

**Published:** 2015-02-03

**Authors:** Eylem Levelt, Stefan K Piechnik, Masliza Mahmod, Vanessa M Ferreira, Rina Ariga, Jane M Francis, Alexander Liu, Joanne Sellwood, Rohan S Wijesurendra, Matthew D Robson, Kieran Clarke, Stefan Neubauer, Theodoros D Karamitsos

**Affiliations:** 1 OCMR, University of Oxford, Oxford, UK; 2 Department of Physiology, Anatomy&Genetics, University of Oxford, Oxford, UK

## Background

Patients with diabetes mellitus (DM) are known to have microvascular coronary artery disease (CAD), in the absence of hypertension and flow limiting stenoses on epicardial coronary arteries. Microvascular coronary dysfunction can be detected on CMR using adenosine stress perfusion imaging.

Native T1 values can detect tissue water content of intra/extracellular or intra/extravascular origin. We hypothesised that increased myocardial blood volume in the intravascular space under adenosine vasodilator stress will have a measurable effect on T1 values. Therefore, we compared adenosine stress T1 response and myocardial perfusion reserve in patients with type-2 DM (T2DM) without macrovascular CAD against controls.

## Methods

27 patients with T2DM (13 male, mean age 54±2years; BMI 28±3) and 24 matched controls (13 male, mean age 51±3 years; BMI 26±1) were studied. Patients were on oral antidiabetic therapies (mean HBA1c 7.44±1.26%) with no known diabetic complications. Obstructive CAD (defined as >50% lumen diameter reduction) was excluded in patients by CT coronary angiography. CMR included cine, rest and adenosine stress native T1-mapping, perfusion imaging (at rest and adenosine stress), late gadolinium enhancement, post-contrast T1-mapping, all performed at 3.0 T. At rest, a mid-ventricular short-axis T1 map was acquired using the Shortened Modified Look-Locker Inversion recovery (ShMOLLI) sequence. Adenosine (140 μg/kg/min) was infused intravenously for 3-6 minutes, and the same mid SA T1 map was acquired at peak stress. followed by first-pass perfusion imaging (0.03 mmol/kg gadoderic acid-Dotarem®). After 20min break rest perfusion imaging was performed. A single mid-ventricular short axis slice was acquired for post-contrast T1 maps 15 minutes after administration of contrast.

## Results

Left ventricular ejection fraction (LVEF) and mass (LVM) were similar in patients with T2DM and controls. During adenosine stress, increases in rate pressure product were similar in patients and controls (T2DM 40±3%, controls 39±3%, p=0.89). Myocardial perfusion reserve index (MPRI) was reduced in patients (1.64±0.08; controls 2.1±0.14, p=0.002) in keeping with microvascular disease. Stress T1 response to adenosine was blunted in patients (controls: from 1189±30ms to 1258±46ms, p<0.001 and patients: from 1193±31ms to 1244±45ms, p<0.001; control DT1=48±35 vs, patient DT1=68±36ms, p=0.05). There was no difference in rest native T1 values (1193±31ms vs. 1189±30ms, P=0.516), or extracellular volume (ECV) measurements (0.29±0.02 vs 0.30±0.03, P=0.112) between groups, suggesting absence of diffuse myocardial fibrosis.

## Conclusions

In patients with T2DM but no significant flow limiting CAD, T1 response under adenosine stress is blunted, likely due to microvascular CAD as suggested by the reduced myocardial perfusion reserve. Adenosine stress native T1 mapping allows the assessment of vascular reactivity, and may be useful particularly for the assessment of patients with contraindications to contrast.

## Funding

The National Institute for Health Research Oxford Biomedical Research Council supported this work.

**Figure 1:**
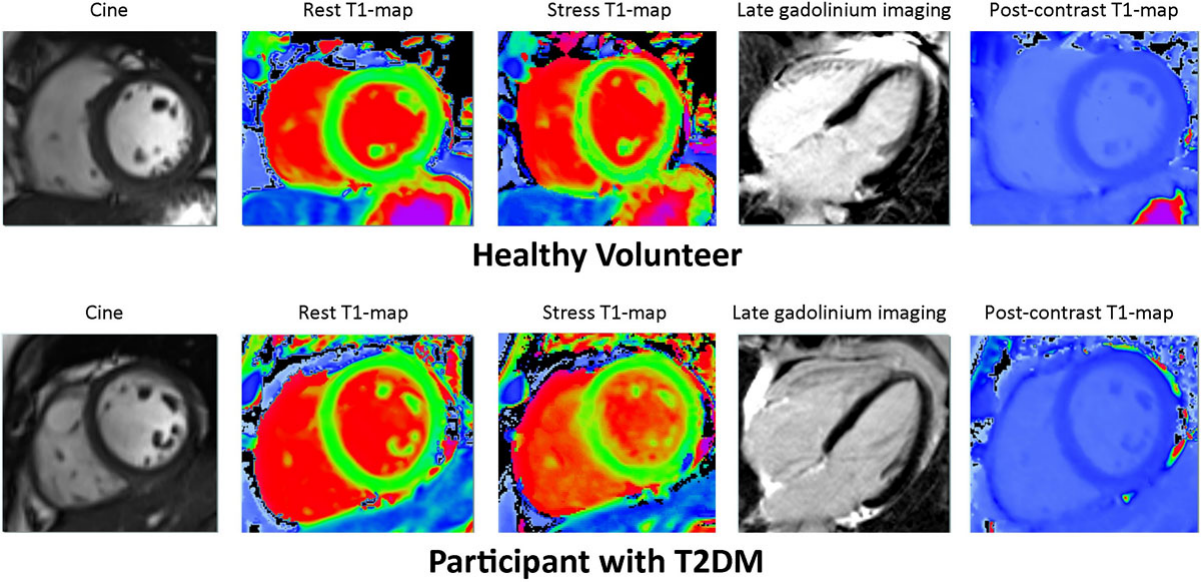


**Figure 2:**